# Slower Dynamics and Aged Mitochondria in Sporadic Alzheimer's Disease

**DOI:** 10.1155/2017/9302761

**Published:** 2017-10-19

**Authors:** Patricia Martín-Maestro, Ricardo Gargini, Esther García, George Perry, Jesús Avila, Vega García-Escudero

**Affiliations:** ^1^Centro de Biología Molecular “Severo Ochoa” (UAM-CSIC), Nicolás Cabrera, 1. Cantoblanco 28049 Madrid, Spain; ^2^Centro de Investigación Biomédica en Red de Enfermedades Neurodegenerativas (CIBERNED), Valderrebollo, 5, 28031 Madrid, Spain; ^3^Brain and Mind Research Institute, Weill Cornell Medical College, Cornell University, 407 E 61st St. 1300 York Avenue, New York, NY 10065, USA; ^4^University of Texas at San Antonio, One UTSA Circle, San Antonio, TX 78249-0667, USA; ^5^Departamento de Anatomía, Histología y Neurociencia, Facultad de Medicina, UAM, Arzobispo Morcillo, 4, 28029 Madrid, Spain

## Abstract

Sporadic Alzheimer's disease corresponds to 95% of cases whose origin is multifactorial and elusive. Mitochondrial dysfunction is a major feature of Alzheimer's pathology, which might be one of the early events that trigger downstream principal events. Here, we show that multiple genes that control mitochondrial homeostasis, including fission and fusion, are downregulated in Alzheimer's patients. Additionally, we demonstrate that some of these dysregulations, such as diminished DLP1 levels and its mitochondrial localization, as well as reduced STOML2 and MFN2 fusion protein levels, take place in fibroblasts from sporadic Alzheimer's disease patients. The analysis of mitochondrial network disruption using CCCP indicates that the patients' fibroblasts exhibit slower dynamics and mitochondrial membrane potential recovery. These defects lead to strong accumulation of aged mitochondria in Alzheimer's fibroblasts. Accordingly, the analysis of autophagy and mitophagy involved genes in the patients demonstrates a downregulation indicating that the recycling mechanism of these aged mitochondria might be impaired. Our data reinforce the idea that mitochondrial dysfunction is one of the key early events of the disease intimately related with aging.

## 1. Introduction

Alzheimer's disease (AD) is a common and devastating dementia that is pathologically defined by the accumulation of extracellular amyloid beta- (A*β*-) containing plaques and intraneuronal hyperphosphorylated Tau protein aggregates associated with neuronal loss in the cerebral cortex. Studies of whole-genome gene expression profiling have identified that AD patients exhibit mitochondrial impairment together with altered calcium signaling and inflammation [1]. Defects in mitochondrial function generate oxidative stress increase due to mitochondrial electron transport chain leakage. The generated reactive oxygen species (ROS) oxidize multiple cell components, being an important issue to trigger protein misfolding [2]. It has been proposed that oxidative stress could be one of the primary events in the development of AD and plays a pivotal role in its pathogenesis [3, 4]. At the same time, mitochondria are targets of ROS causing the oxidation of their components increasing mitochondrial deterioration. This mitochondrial dysfunction may be one of the earliest and most prominent features of AD [5, 6]. Several works that show downregulation of mitochondrial genes in Alzheimer's have also demonstrated a reduced metabolic rate in the brain of these patients measured by fluorodeoxyglucose PET [7]. Metabolic abnormalities besides the damage of both components and the structure of mitochondria are well described in AD [8–10]. Moreover, several models have revealed that mitochondrial dysfunction triggers the aberrant processing of APP and tau [11–15]. Another deregulated aspect in AD is calcium signaling, which is closely related to functional the status of mitochondria. Experimental models have demonstrated that mitochondrial dysfunction favors tau phosphorylation, microtubule depolymerization, and neurofibrillary tangle-like pathology [11]. Hippocampal neurons exhibited AD-like tau phosphorylation and high calcium levels due to glutamate exposition impairing mitochondrial function [16].

Mitochondria are dynamic organelles that continuously undergo fission and fusion events which are necessary for cell survival as well as adaptation to changing conditions needed for cell growth, division and morphology, and distribution of mitochondria [17]. Mitochondrial dynamics is regulated by a machinery involving large dynamin-related GTPases where mitofusin 1 (MFN1), mitofusin 2 (MFN2), and optic atrophy 1 (OPA1) are involved in mitochondrial fusion [18], whereas mitochondrial fission is mediated by dynamin-like protein 1 (DLP1) through the interaction with four mitochondrial receptor proteins [18–20]. Dynamic fusion and fission processes allow damaged mitochondria to be recycled by a degradation mechanism termed mitophagy [21]. This pathway is partially driven by PINK1 and PARK2. When mitochondria are damaged and membrane potential is lost, there is a rapid recruitment of PARK2 to the mitochondria mediated by PINK1 that promotes K63-linked ubiquitin chain signaling [22]. Adaptor proteins of the autophagic system that recognize the ubiquitinated cargo through ubiquitin-binding domains (UBDs) and microtubule-associated proteins 1A/1B light chain 3 (LC3)/GABAA receptor-associated protein (GABARAP) attach to autophagosomal membranes via an LC3-interacting region (LIR) [22]. The autophagy machinery promotes the ubiquitinated cargo engulfment by autophagosomes and its final degradation [23].

In AD, mitochondrial failure might arise from a deficient dynamic balance of mitochondrial fission and fusion that is greatly shifted toward fission, and it may result in the presence of dysfunctional mitochondria in damaged neurons as well as fibroblasts from AD patients characterized by their accumulation into the perinuclear areas [24, 25]. In addition, autophagy dysfunction in brain and peripheral tissues of AD patients is widely documented [26]. This has been mainly associated to reduce degradative function, insufficient lysosomal pH acidification, and low hydrolases activity [27, 28] impairing the recycling of damaged mitochondria and generating a mitophagy failure [26].

In the present work, we have demonstrated a downregulation of genes involved in mitochondrial dynamics as well as in autophagy and mitophagy in Alzheimer's patients. Consequently, we have found slower mitochondrial dynamics correlating with diminished STOML2, MFN2, and DLP1 levels, showing the last one reduced mitochondrial localization, and therefore causing the accumulation of aged mitochondria in fibroblasts from AD patients.

## 2. Materials and Methods

### 2.1. Primary Cells and Culture Conditions

Primary skin fibroblasts were obtained from Coriell Institute for Medical Research (NJ, USA). Five fibroblast cell lines from SAD patients and five correspondent apparently healthy sex- and age-matched samples have been used (see Table 1 for details about age, sex, and stage of the disease). Human fibroblasts were cultured in Dulbecco's modified Eagle's medium (DMEM) supplemented with 10% (*v*/*v*) heat-inactivated fetal bovine serum (FBS), 2 mM glutamine, 10 U/ml penicillin, and 10 *μ*g/ml streptomycin, in 5% CO_2_ in a humid incubator at 37°C. The use of fibroblasts has been restricted to a maximum of 10 cell passages to avoid replicative senescence, and cultures were always kept below confluence.

### 2.2. Microarray Analysis

The analysis of specific gene expression profiling by array (Affymetrix Human Gene 1.0 ST Array) of DLP1, GDAP1, MIEF1, MFN2, OPA1, STOML2, Optineurin, ATG5, ATG12, Beclin1, PI3K class III, ULK1, AMBRA1, BNIP3, BNIP3L, FUNDC1, VDAC1, and VPC/p97 of human brain samples was retrieved from Berchtold data set [29]. This study contains 253 samples from 84 patients, of which 173 are samples of different brain zones of 56 healthy subjects (aged 20–99 years) and 80 are samples of different brain zones of 28 AD patients (aged 28–99 years). Microarray data were obtained from 4 brain regions: the hippocampus, entorhinal cortex, superior frontal cortex, and postcentral gyrus. A similar analysis was performed in an additional data set of human brain samples classified into healthy (*n* = 47) versus AD (*n* = 32) patients obtained from the Hisayama study [30]. Differences in gene expression between healthy and AD patients were calculated using Student's *t*-test.

### 2.3. Antibodies

The primary antibodies used were TOMM20 (sc-11415), MFN2 (sc-515647), and STOML2 (sc-376165) and were purchased from Santa Cruz Biotechnology, Santa Cruz, CA, USA; DLP1 (611112, BD Biosciences); GAPDH (ab8245, Abcam, Cambridge, UK); and *β*-tubulin (T4026, Sigma-Aldrich). The secondary antibodies for Western blot studies were horseradish peroxidase-conjugated antimouse IgGs (P0161, DAKO) and for immunofluorescence were antirabbit IgGs alexa-488 or antimouse alexa-555 labelled (Molecular Probes, Millipore, Waltham, MA).

### 2.4. Western Blot Analysis

The cells and tissue samples were homogenized in lysis buffer (50 mM pH 7.5 HCl-Tris, 300 mM NaCl, 0.5% SDS, and 1% Triton X-100) and incubated 15 min at 95°C. Protein concentration of the extracts was measured using the DC protein assay kit (500–0111, Bio-Rad). Equal amounts of total protein extract from healthy and SAD cells were resolved by SDS-PAGE and then transferred to nitrocellulose (G9917809, Amersham, Germany) or PVDF (IPVH00010, Merk Millipore, Cork, Ireland) membranes. Western blot and immunoreactive proteins were developed using an enhanced chemiluminescence detection kit (NEL105001EA, Perkin Elmer) following instructions of the supplier. Quantification was performed by densitometry of the obtained bands in each lane with respect to the correspondent housekeeping protein such as GAPDH or *β*-tubulin band in each experiment (Quantity One software, Bio-Rad).

### 2.5. Lentivirus Production

Pseudotyped lentivectors were produced using reagents and protocols from Prof. Didier Trono. Briefly, viral stocks were produced by transient cotransfection of 293 T cells with 5 *μ*g of the corresponding lentivector plasmid, 5 *μ*g of the packaging plasmid pCMVdR8.74 (Addgene plasmid 22036) and 2 *μ*g of the VSV-G envelope protein plasmid pMD2G (Addgene plasmid 12259) using Lipofectamine and Plus reagents following instructions of the supplier (18324 and 11514, resp., Life Technologies). Lentiviral titer was calculated by infecting cells with increasing amounts of lentiviral supernatant, and the percentage of infected cells was detected by flow cytometry (FACSCalibur, BD Biosciences, San Jose, CA).

### 2.6. Mitochondrial Dynamics Study

Cells were infected at an approximate multiplicity of infection (MOI) of 5–10 with a lentivector encoding DsRed2-Mito construct obtained from Clontech (632421) that was kindly provided by Dr. Ismael Santa-María (Columbia University, NY). Cells were treated with 20 *μ*M carbonyl cyanide m-chlorophenylhydrazone (CCCP, Sigma, St. Louis Missouri) for 6 h and, in the reversible condition, CCCP was removed after 1 h and the medium was replaced for DMEM 10% FCS. After the treatments cells were fixed with 4% paraformaldehyde (PFA) and the mitochondrial pattern was observed with an Axioskop2 plus microscope coupled to a CCD color camera (Zeiss). For analysis, we used the Mitochondrial Network Analysis (MiNa) toolset, a combination of different ImageJ macros that allows the semiautomated analysis of mitochondrial networks in cultured mammalian cells [31]. Briefly, the image was converted to binary by thresholding following the conversion to a skeleton that represents the features in the original image using a wireframe of lines of one pixel wide. All pixels within a skeleton were then grouped into three categories: end point pixels, slab pixels, and junction pixels. The plugin analyzes how the pixels are spatially related and defined to measure the length of each branch and the number of branches in each skeletonized feature as well as the mitochondrial network morphology. The parameters used in the study were (1) individuals, punctate, rods, and large/round mitochondrial structures; (2) networks, mitochondrial structures with at least a single node and three branches; (3) the mean number of branches per network; and (4) the average of length of rods/branches. Ten randomly chosen fields containing between 10 and 15 cells were used to quantify the pattern of mitochondria. We classify the mitochondrial morphology into three different subtypes according to the length of the branches: filamentous (long and spaghetti-like shape; branch > 2.3 *μ*m), fragmented (completely dotted; branch < 1.8 *μ*m), and intermediate pattern (when both filamentous and fragmented mitochondria were found; 1.8 *μ*m ≥ branch ≥ 2.3 *μ*m).

### 2.7. Immunocytochemistry

Fibroblasts were grown on sterile glass coverslips, treated as described for each experiment, followed by washing with PBS and fixing with 4% PFA in PBS for 30 min at room temperature. After blocking with PBS containing 1% BSA and permeabilizing with 0.1% Triton X-100 and 1 M glycine for 30 minutes, cells were then stained with DLP1 and TOMM20 antibody diluted according to the manufacturer's recommendation in blocking buffer overnight at 4°C. Cells were washed with PBS and stained with secondary antibody (1 : 500 in blocking buffer) for 2 h at room temperature. Samples were mounted with ProlongGold Antifade (P-36930, Life Technologies) and randomly chosen field images were obtained in an Invert Confocal LSM510 (Zeiss, Oberkochen, Germany) fluorescence microscope.

### 2.8. Colocalization Study

Colocalization analysis was performed with ImageJ software (Bethesda, MD). The background of different channels was edited with the subtract background tool with a rolling ball radius of 30 pixels, and binary images were obtained by a threshold intensity. The logical operation AND of the Image Calculator tool was used to generate an image harboring only overlapping structures of both channels. Colocalization measurement was obtained by quantifying the area occupied by the overlapping elements per cell. At least 200 cells were measured for each cell line.

### 2.9. Mitochondrial Potential Measurement

After treatment, fibroblasts were incubated with 200 nM TMRM (T668, Molecular Probes, Waltham, MA, USA) for 30 min at 37°C and analyzed by flow cytometry (FACSCalibur, BD Biosciences). Mitochondrial membrane potential for each condition was represented as the percentage of the fluorescence intensity mean with respect to the fluorescence intensity mean exhibited when these cells remained untreated.

### 2.10. Study of Mitochondrial Age

MitoTimer transgene was amplified by PCR from the plasmid pTRE-Tight-MitoTimer (Plasmid number 50547, Addgene, MA, USA) using the oligos: CCTGGAGAATTCAGATCTCCAC and GATCCTGATCACTACAGGAACAGGTGGTGGC, and amplicon was cloned into lentiviral construct pLVX-TetOne-Puro (631849, Clontech, CA, USA) by restriction sites EcoRI and BclI. Fibroblasts were infected by packaged lentiviral particles at an approximated MOI of 5–10. Forty-eight hours postinfection, the expression of the transgene was induced with 2 *μ*g/ml of doxycycline during 24 h and then removed from the media to analyze it at different times. On the other hand, after 24 h of doxycycline, cells were treated with CCCP (20 *μ*M) for 24 h and then removed from media to be analyzed. Red and green fluorescence intensity was followed over time by flow cytometry (FACSCalibur, BD Biosciences). Aged mitochondrial quantification was calculated as the percentage of red fluorescence intensity mean at each point with respect to the one of the untreated cells at the beginning of the study (doxycycline removal, 0 h).

### 2.11. Statistical Analysis

Graphs represent means and standard deviations of the values obtained from experimental triplicates. When necessary, values represented in the graphs were obtained by normalizing every SAD sample data with its correspondent age-matched healthy sample. Statistical comparison of the data sets was performed by the Student's *t*-test. Two-way analysis of variance test was performed to examine the differences between experimental factors and their interaction. A post hoc Bonferroni test was used when more than two experimental groups were compared.

## 3. Results

### 3.1. Gene Expression Alteration of Mitochondrial Dynamics Proteins in AD Brains

**Figure 1:**
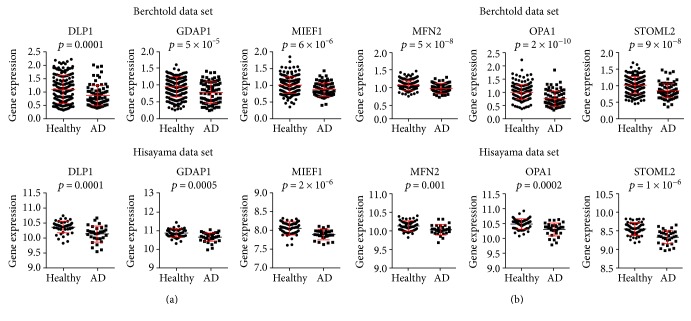
Downregulation of dynamics genes in AD brains. (a)-(b) Analysis of microarray expression in two independent data sets of genes involved in mitochondrial fission (a) and fusion (b). Graphs show dot plots of the expression of the indicated genes using in upper row *n* = 253 samples from 84 patients, of which *n* = 56 are healthy subjects and *n* = 28 are AD patients retrieved from Berchtold data set [29] and in lower row *n* = 47 controls and *n* = 32 AD brain samples from the Hisayama study data set [30]. Correspondent *p* value was determined by Student's *t*-test and is showed for each graph.

Mitochondrial function disruption has been proposed long time ago as an early alteration in Alzheimer's pathology [32]. Mitochondria are a dynamic tubular network that undergo fission and fusion as a fundamental process for mitochondrial renewal and elimination of damaged ones [21]. Here, we observed that several fission proteins such as DLP1, GADP1, and MIEF1 appeared diminished in AD brain samples in Berchtold et al. [29] and Hokama et al. [30] data sets (Figure 1(a)). Multiple works have shown DLP1 is recruited to mitochondria to constrict the membranes upon assembly into spirals and to induce the scission of mitochondria upon GTP hydrolysis [33]. Membrane stabilization of DLP1 is regulated by GADP1 and MIEF1 [33]. On the other hand, fusion proteins such as MFN2, OPA1, and STOML2 have been shown diminished in Alzheimer's brain in both data sets (Figure 1(b)). Fusion is coordinated by MFN1/2 and OPA1, where the first one catalyzes the fusion of external membrane and the second one is associated to the internal membrane [34, 35]. An additional system named hyperfusion is controlled by STOML2/OPA1, which is stimulated under stress conditions favoring cell survival [36]. In summary, we could observe that both fission and fusion processes were reduced in AD brain accordingly with the mitochondrial dysfunction that takes place in this pathology.

### 3.2. Downregulation of Mitochondrial Dynamics Proteins in SAD Fibroblasts

**Figure 2:**
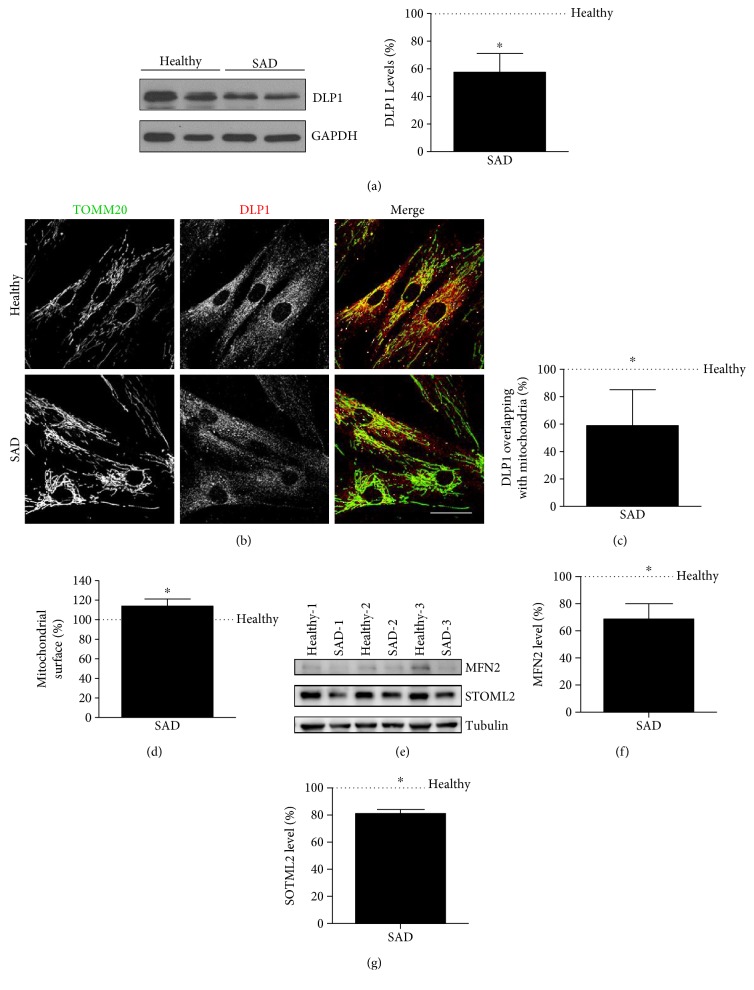
Decreased fission and fusion protein levels in SAD cells. (a) Representative Western blot analysis and quantification of DLP1 in healthy and SAD fibroblasts under basal conditions. (b) Representative confocal microscopy images showing TOMM20 in green and DLP1 in red in basal conditions. (c) Quantification of the amount of DLP1 in mitochondria per cell in the images represented in (b). (d) Quantification of mitochondrial surface measured by TOMM20 label per cell in the images represented in (b). (e)–(g) Representative Western blot analysis and quantification of MFN2/mitofusin 2 (f) and STOML2 (g) in healthy and SAD fibroblasts under basal conditions. Graphs show means and standard deviations of the healthy/AD age- and sex-matched couple samples: AG11020/AG05810 and AG11362/AG05809; AG07310/AG06869; AG05813/AG06263; AG07803/AG06262 (a)–(e) and AG11020/AG05810; AG11362/AG05809; and AG07310/AG06869 (g). Scale bar: 40 *μ*m. ^∗^*p* < 0.05.

Taking into account the previous results, we next examined whether proteins that control mitochondrial dynamics could be altered in patient-derived peripheral cells such as fibroblasts. As several works have shown reduced levels of DLP1 in neurons as well as in fibroblasts from sporadic AD patients [37, 38], we focused our attention in this protein. We could observe a decrease in DLP1 fission protein level, in SAD cells as it was previously described by Zhu and coworkers [38] (Figure 2(a)). DLP1 function depends on its subcellular localization [39]; therefore, we studied its distribution by immunofluorescence analysis of mitochondria labeled with TOMM20 (Figure 2(b)). Quantitative analysis revealed a decrease of DLP1 on mitochondria in SAD fibroblasts compared to healthy ones (Figure 2(c)). Additionally, the mitochondrial surface was increased in SAD fibroblasts with respect to the healthy ones (Figure 2(d)), as we have previously reported [26]. Finally, as it has been described for patients' brain [40], we have also observed diminished fusion protein levels such as MFN2/mitofusin2 and STOML2 in SAD fibroblasts (Figures 2(e), 2(f), and 2(g)). These data indicate that the putative mitochondrial defect associated to AD may be also found in fibroblasts.

### 3.3. Mitochondrial Dynamics Alteration in SAD Fibroblasts

**Figure 3:**
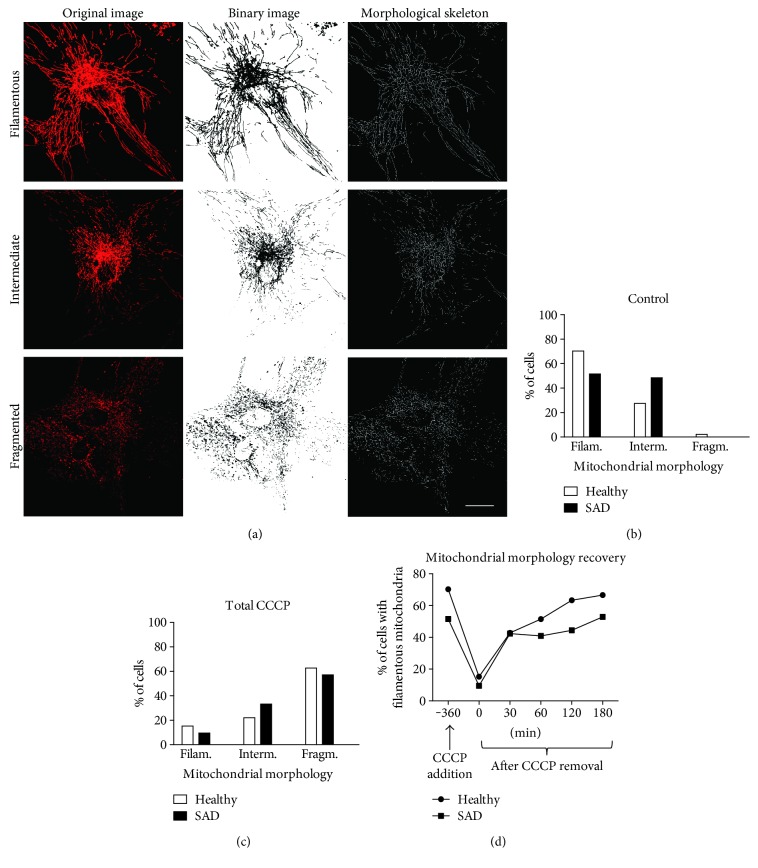
Mitochondrial dynamics alterations in SAD fibroblasts. (a) Representative confocal microscopy images showing the three different mitochondrial morphologies in which they will be classified followed by binary image and the subsequently morphological skeleton generated by Mitochondrial Network Analysis (MiNa) toolset [31]. (b)–(c) Quantification of mitochondrial morphology of healthy and SAD couple under basal conditions (b) and treated with 20 *μ*M CCCP for 7 h (c). (d) Time course of the mitochondrial morphology recovery in healthy and AD fibroblasts reversibly treated with CCCP. Mitochondrial morphology classification according to the length of the branches in filamentous (branch > 2.3 *μ*m), fragmented (branch < 1.8 *μ*m), and intermediate (1.8 ≥ branch ≥ 2.3 *μ*m) patterns. Graphs represent the data of AG11362/AG05809 healthy/AD age- and sex-matched couple. Scale bar: 40 *μ*m.

Abnormal mitochondrial distribution and morphology have been described in SAD fibroblasts [38]. These data together with the downregulation of mitochondrial dynamics proteins previously described led us to conclude that mitochondrial dynamics may be altered in SAD fibroblasts. To seek for a possible functional defect in mitochondrial dynamics, we studied a reversible process of mitochondrial fragmentation. Healthy and SAD cells, which had similar growth rates, were plated and infected with a lentivector encoding Mito-DsRed2 for better mitochondria visualization and treated with carbonyl cyanide m-chlorophenylhydrazone (CCCP). These conditions induce a reversible depolarization of mitochondria without causing any toxic effect, and the viability of fibroblasts was not compromised (data not shown). The analysis of the images was done by the Mitochondrial Network Analysis (MiNa) toolset [31]. The patterns of mitochondria were classified into three categories according to the length of the branches: filamentous, fragmented, and intermediate (Figure 3(a); see Materials and Methods). Analysis was carried out by the quantification of mitochondrial shape in all the different experimental conditions. SAD and healthy cells exhibited similar mitochondrial pattern under resting conditions (Figure 3(b)) where most cells showed a filamentous and intermediate morphology. Conversely, the mitochondrial morphology was mainly fragmented when we treated the cells with CCCP for 7 h (Figure 3(c)).

**Figure 4:**
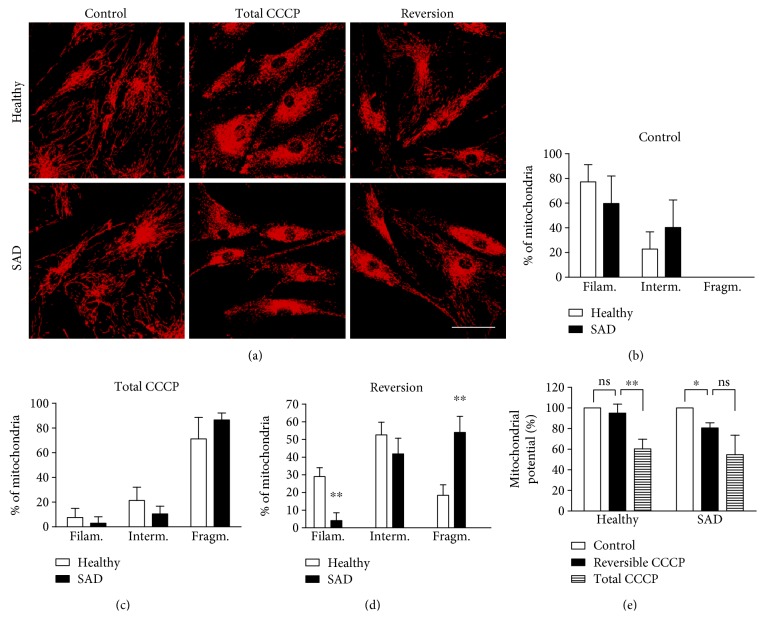
Delayed recovery of mitochondrial filamentous morphology in SAD fibroblasts. (a) Representative confocal microscopy images showing the mitochondrial morphology of healthy and SAD fibroblasts untreated (control), after 7 h with 20 *μ*M CCCP (total CCCP) or after 6 h of CCCP treatment followed by 1 h without it (reversion). (b)–(d) Quantification of mitochondrial morphology of healthy-SAD couples when the cells remain untreated (b), treated with CCCP for 7 h (c), or reversibly treated with CCCP (d). (e) Mitochondrial membrane potential measured by TMRM of fibroblasts treated as in (a). Mitochondrial morphology classification according the length of the branches in filamentous (branch > 2.3 *μ*m), fragmented (branch < 1.8 *μ*m), and intermediate (1.8 ≥ branch ≥ 2.3 *μ*m) patterns. *n* = 3 different healthy/AD sex- and age-matched couple samples: AG11362/AG05809, AG07310/AG06869, and AG11362/AG05809. Scale bar: 40 *μ*m. ^∗^*p* < 0.05 and ^∗∗^*p* < 0.01; ns: not significant.

Although there were no substantial differences in the static pictures before and after CCCP treatment between healthy and SAD fibroblasts, we sought for a possible alteration during the process of fragmentation or fusion as a consequence of the dynamic proteins' downregulation as described. With this aim, we carried out a time course analysis of the mitochondrial morphology recovery over the time. Thus, fibroblasts were treated with CCCP for 6 h (*t* = 0 h); afterwards, they were allowed to recover during different time points (Figure 3(d)). We saw that after CCCP removal, there was a rapid initial recovery of filamentous morphology of both healthy and SAD cells within the first 30 min. However, after this time point, there was no recovery of filamentous morphology in SAD cells in the following 30 min, contrary to healthy fibroblasts that exhibited a quicker increase of filamentous mitochondria. Moreover, we observed that both healthy and SAD fibroblasts recovered the filamentous morphology over the time up to the percentages of untreated cells (Figure 3(d), −360 min). Thus, we chose 1 h of recovery after CCCP treatment to analyze the mitochondrial morphology recovery in all the control/SAD fibroblasts couples used in this work (Figures 4(a), 4(b), 4(c), and 4(d)). As before, healthy and SAD cells exhibited similar mitochondrial pattern under resting conditions (Figures 4(a) and 4(b)) or after 7 h of CCCP treatment (Figures 4(a) and 4(c)). However, after the reversible challenge with CCCP, SAD fibroblasts exhibited significantly increased percentage of cells with fragmented mitochondria with respect to their correspondent healthy fibroblasts, which showed a significantly higher proportion of cells that had recovered filamentous morphology (Figures 4(a) and 4(d)). Additionally, we could observe that CCCP treatment induced similar mitochondrial potential membrane (Δ*Ψ*m) loss in healthy and SAD fibroblasts (Figure 4(e)). However, when these cells were allowed to recover, only healthy fibroblasts recovered Δ*Ψ*m up to the levels of untreated cells (Figure 4(e)). On the contrary, SAD fibroblasts although some increase of Δ*Ψ*m was observed indicating that cells tend to recover, there were no significant differences with respect to total CCCP condition and still maintaining significant differences with respect to untreated condition (Figure 4(e)). These results suggest a delayed recovery of mitochondrial filamentous morphology and membrane potential in SAD fibroblasts after a reversible insult.

**Figure 5:**
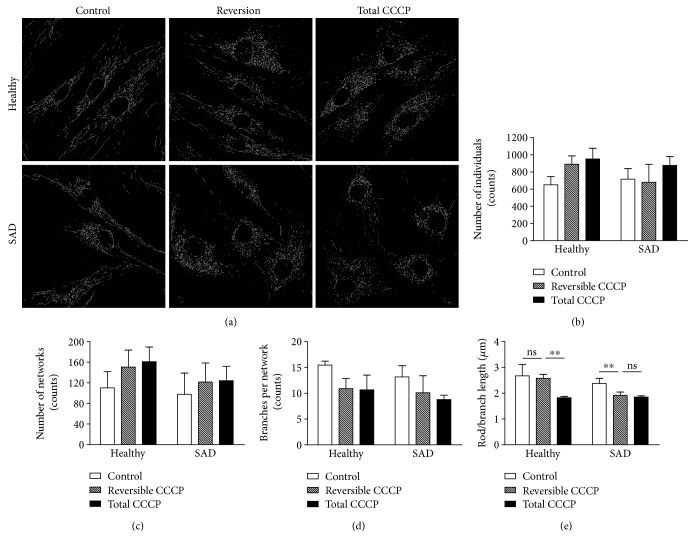
Analysis of mitochondria network. (a) Representative images showing mitochondrial morphological skeleton obtained with MiNa toolset [31] of healthy and SAD fibroblasts untreated (control), after 6 h of CCCP treatment followed by 1 h without it (reversion) or after 7 h with 20 *μ*M CCCP (total CCCP). (b)–(e) Quantification of number of individuals (b), networks (c), branches per network (d), or length of the branches (e) of *n* = 3 different healthy/AD sex- and age-matched couple samples: AG11362/AG05809, AG07310/AG06869, and AG11362/AG05809. ^∗∗^*p* < 0.01; ns: not significant.

Then, we moved further into the morphological features of mitochondria to study in detail the mitochondrial network. We examined the mitochondrial network skeleton in representative images of the three experimental conditions (control, reversion and total CCCP) in both healthy and SAD fibroblasts, and the network parameter values were then calculated (Figure 5(a)). Although, there were no differences in the number of individuals (puncta and rods mitochondrial structures, Figure 5(b)) as well as networks (mitochondrial structures with at least a single node and three branches, Figure 5(c)) between healthy and SAD cells, we observed that the treatment with CCCP increased the number of individuals and networks in both cases (Figures 5(b) and 5(c)). The augmented number of networks was related to the fragmentation of larger networks with more branches into many smaller networks as it was reflected by the decreased number of branches per network after the treatment with CCCP (Figure 5(d)). Additionally, we analyzed the length of the branches in each condition (Figure 5(e)). As expected, we saw that the branch length was remarkably diminished after the CCCP treatment in both cells lines (Figure 5(e)). Surprisingly, we noticed that after 1 h of recovery without CCCP, the length of the branches in SAD cells was the same than after 7 h of CCCP. In contrast, healthy fibroblasts exhibited a recovery of the branch length up to the values of the untreated condition, suggesting once again a delayed recovery of the mitochondrial network in SAD cells (Figure 5(e)).

### 3.4. Increased Mitochondrial Aging in SAD Fibroblasts due to Recycling Impairment

**Figure 6:**
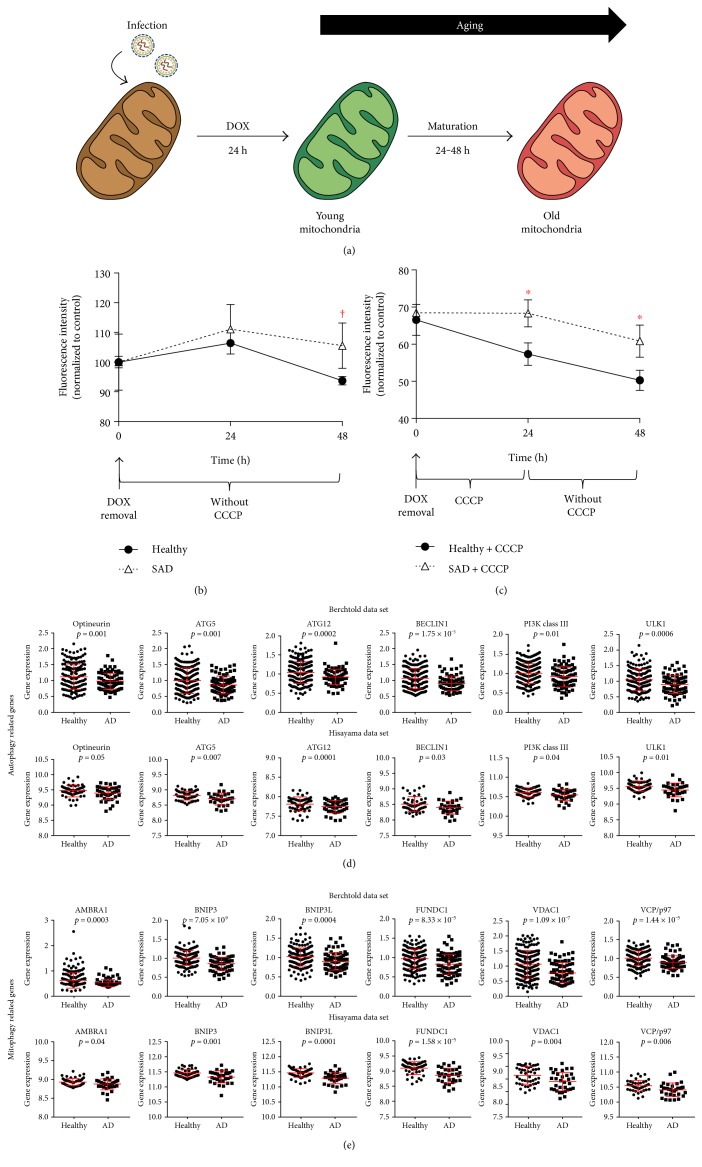
Aged mitochondria due to defective mitochondrial recycling in AD. (a) Scheme of the function of lentiviral MitoTimer tool. (b) Graph showing intensity of red fluorescence per cell measured by flow cytometry over timer in healthy and SAD fibroblasts previously infected by a lentivirus encoding MitoTimer and treated for 24 h with doxycycline. (c) Graph representing a similar study but in the presence of CCCP (20 *μ*M) for 24 h after doxycycline removal. (b)–(c) Values represent means and standard deviations of the percentage of red fluorescence intensity mean per cell (aged mitochondria) with respect to control condition that is the average of red fluorescence intensity mean after doxycycline removal without CCCP (0 h time point) (*n* = 3 independent experiments using the healthy/SAD couple AG01362/AG05859, †*p* < 0.08, and ^∗^*p* < 0.05). (d)–(e). Analysis of microarray expression of genes involved in autophagy (d) and mitophagy (e). Graphs show dot plots of the expression of the indicated genes using in upper rows *n* = 253 samples from 84 patients, of which *n* = 56 are healthy subjects and *n* = 28 are AD patients retrieved from Berchtold data set [29], and in lower rows *n* = 47 controls and *n* = 32 AD brain samples retrieved from the Hisayama study [13]. Correspondent *p* value was determined by Student's *t*-test and is showed for each graph.

To investigate whether the mitochondrial dynamics failure could affect the quality of the mitochondria, we analyzed the mitochondrial turnover in healthy and SAD fibroblasts by using the MitoTimer tool. With this aim, we cloned the MitoTimer gene [41] into a lentiviral vector that encodes all the regulatory elements to allow transgene expression activation in response to doxycycline, pLVX-TetOne-Puro (631849, Clontech, CA, USA). MitoTimer provides a fluorescent readout which directly relates to the mitochondrial turnover and quality control described in several studies based on a time-sensitive fluorescent protein [41–43]. It consists of the timer fluorescent protein fused to the mitochondrial targeting sequence of COX8A subunit, which targets MitoTimer to the mitochondrial matrix. Timer, initially fluorescence green, changes over time so the emission shifts from green to red. Healthy and SAD fibroblasts were infected with the lentivirus encoding MitoTimer and, after 48 h, transgene expression was induced by doxycycline treatment for 24 h, after what CCCP was added during 24 h (Figure 6(a)). We monitored by flow cytometry the amount of aged mitochondria measured by red fluorescence. After doxycycline removal, aged mitochondria were increased similarly in both healthy and SAD fibroblasts during the first 24 h (Figure 6(b), 24 h). However, the recycling of these aged mitochondria was slightly faster in healthy cells with respect to SAD ones (Figure 6(b), 48 h). When we boosted mitochondrial recycling process by the addition of CCCP, we could observe that aged mitochondria were diminished in healthy fibroblasts due to an efficient degradation of dysfunctional mitochondria (Figure 6(c), 24 h healthy). Meanwhile, in SAD fibroblasts, there was no degradation of aged mitochondria during the treatment with CCCP (Figure 6(c), 24 h SAD). After removal of CCCP, there were still significant differences in the amount of aged mitochondria between healthy and SAD fibroblasts (Figure 6(c), 48 h), correlating with our previous results of a slower mitochondrial dynamics and membrane potential recovery. Our results indicate that SAD fibroblasts exhibited aged mitochondria compared to healthy cells and the recycling process was impaired. This result correlates with our previous work in which we have demonstrated a functional impairment of autophagy and, more specifically, of mitophagy in this set of SAD fibroblasts [26].

### 3.5. Alzheimer's Disease Patients Exhibit Reduced Expression of Autophagy and Mitophagy-Associated Genes

Numerous works have focused their efforts in the study of mitochondrial recycling mechanisms due to their relevance in aging [44]. Mitochondrial fusion and fission processes are closely related with the removal of damaged mitochondria executed by mitophagy. Due to the fact that we have observed the accumulation of aged mitochondria in SAD fibroblasts, we wondered whether autophagy and mitophagy genes may be altered in the two sets of samples where we previously observed a downregulation of mitochondrial dynamics genes. We observed that a high number of autophagy associated genes such as Optineurin, ATG5, ATG12, Beclin1, PI3K class III, and ULK1 (Figure 6(d)) and mitophagy such as AMBRA1, BNIP3, BNIP3L, FUNDC1, VDAC1, and VCP/P97 (Figure 6(e)) were downregulated in AD brain with respect to healthy controls.

## 4. Discussion

### 4.1. Alteration of the Expression of Fission and Fusion-Related Genes

Alzheimer's is the most prevalent neurodegenerative disease and is characterized by progressive dementia that initiates as short-term memory impairment. In the past decade, a wide number of gene profiling analysis have been performed in patient-derived postmortem brains as well as peripheral tissues. These studies have revealed a narrow relationship of mitochondrial dysfunction, calcium signaling, and neuroinflammation with AD pathology [1]. Particularly interesting is the involvement of mitochondria in AD, in fact, several lines of evidence propose mitochondrial anomalies as one of the primary factors that lead to late-onset SAD pathology [9, 45]. Here, we analyzed gene profiling data of genes involved in mitochondrial dynamics and we could observe a pleiotropic downregulation of gene expression of both fission and fusion processes, correlative to other studies in which diminished levels of these proteins have been demonstrated in patients' brain [40]. Accordingly, we have shown that SAD fibroblasts exhibited lower levels of proteins involved in fission-like DLP1 and fusion such as MFN2 and STOML2. Other authors have shown that mitochondrial failure may arise from a deficient dynamic balance of mitochondrial fission and fusion that, in AD, is greatly shifted toward fission, due to a defect in fusion. This may result in the presence of dysfunctional mitochondria in damaged neurons as well as fibroblasts from AD patients characterized by their accumulation into perinuclear areas [24, 25]. We confirmed the downregulation of several fission genes, but also important fusion genes such as MFN2 and OPA1 appeared downregulated, what had been demonstrated to inhibit this process [46, 47]. Furthermore, fusion and fission proteins have been found diminished in fibroblasts suggesting that both processes are affected.

### 4.2. Downregulation of Fusion and Fission Genes Lead to Slower Dynamics

Vulnerable neurons in AD's brain exhibit significant reduction in mitochondrial length and increased width with a significant augmented overall size consistent with unopposed fission suggesting alterations of mitochondrial dynamics [38, 48]. In agreement with these findings, abnormal distribution of mitochondria was found in pyramidal neurons of AD-affected individuals where mitochondria were redistributed away from axons [40]. Accordingly, levels of fusion proteins OPA1, MFN1, and MFN2 were significantly reduced, whereas levels of Fis1 were significantly increased in AD [40]. Primary hippocampal neurons treated with A*β*-derived diffusible ligands (ADDLs) demonstrated shortened mitochondria in neurons and alteration of fission and fusion proteins [40]. Moreover, time-lapse recordings in these neurons showed impairment of both fission and fusion processes, with fusion being more severely affected [40]. These authors discuss that the observed imbalance of fusion and fission proteins does not explain the observed time-lapse recordings where both processes are affected. This last result correlates with our observations of slower mitochondrial dynamics after the addition and removal of CCCP.

Abnormal mitochondrial morphology had been previously found in fibroblasts from sporadic AD patients, where they become significantly elongated and form a highly connected network [38]. However, in our hands, we could not detect significant differences in the number of individuals, networks, branches per network, or length of the branches of mitochondria between healthy and SAD fibroblasts. Only increased mitochondrial surface was detected in SAD fibroblasts. This discrepancy in mitochondrial morphology with the data reported in neurons may be due to variations in the expression pattern of proteins involved in dynamics as well as different mitochondrial function requirements between both cell types. As we have seen a pleiotropic downregulation of fusion and fission genes in brains, our functional study in fibroblasts was focused in both processes. Accordingly, we could observe that the whole dynamics system was affected.

The alteration in mitochondrial dynamics leads to severe consequences in the cell such as structural changes in the cristae formation and assembly of electron transport complex compromising bioenergetics and causing calcium dyshomeostasis, increased oxidative stress, mtDNA damage, and synaptic dysfunction [25].

In addition to the defect observed in fusion/fission, defects in mitochondrial mobility have also been observed in AD causing a mitochondrial reduction in neurites [25]. A*β* induces a reduction in motile mitochondria [49], and ADDLs impair anterograde and retrograde axonal transport of mitochondria in hippocampal neurons [50]. We have also observed the downregulation of many genes involved in mitochondrial transport such as the family of dynactins (DCTN1, 2, 3, 5, and 6), dyneins (DYNC1H1, DYNC1I1, and DYNC1LI1), kinesins (KIF1B and KIF5C), and Miro (data not shown). It has been described that PINK1 and PARK2 target Miro for phosphorylation and degradation to arrest mitochondrial motility keeping in quarantine damaged mitochondria prior to their clearance by mitophagy [51]. Therefore, Miro downregulation may affect to the correct segregation of dysfunctional mitochondria and its subsequently recycling.

### 4.3. Autophagy/Mitophagy Defect and the Resulting Accumulation of Damaged Mitochondria

Numerous works have demonstrated that autophagy is dysregulated in AD pathology, even when autophagy induction has been considered a therapeutic target [52]. More recently, we have reported mitophagy impairment in these patients' brain and peripheral tissue due to insufficient generation of autophagic vesicles together with deficient PARK2 signaling, leading to the accumulation of damaged mitochondria, strong oxidative damage, and accumulation of polyubiquitinated proteins [26]. Accordingly, we now report the downregulation of multiple genes involved in autophagy and mitophagy that was accompanied by the accumulation of aged mitochondria. For this study, we have developed a new lentiviral vector that allows the induction of MitoTimer tool [41, 53] under the control of doxycycline. Our vector has the ability to infect any kind of cell independently of its proliferative state and through a single virus infection achieves the complete regulation of the transgene. This tool will allow the study of mitochondrial aging as well as recycling in vitro and in vivo in any cell or animal model.

## 5. Conclusions

Here, we report numerous data that reveal a deep deregulation of mitochondrial homeostasis in Alzheimer's disease. We have observed a downregulation of mitochondrial fusion and fission in the disease. Accordingly, mitochondrial dynamics is slowed down in these patients' fibroblasts after an insult. This is accompanied by a downregulation of autophagy and mitophagy genes together with the accumulation of aged mitochondria which may be a cornerstone of Alzheimer's pathology due to their involvement in ROS production, elevated calcium levels, and neuroinflammation observed in these patients. Our data continue in fueling the hypothesis that AD is an acceleration of aging caused by malfunction of recycling.

## Figures and Tables

**Table 1:** Core set of fibroblast cell lines.

Line	Age/sex	Clinical diagnosis
AG11362	63/F	Nonaffected
AG05813	67/F	Nonaffected
AG07803	66/M	Nonaffected
AG07310	60/F	Nonaffected
AG11020	79/F	Nonaffected
AG05809	63/F	Moderate dementia
AG06263	67/F	Moderate dementia
AG06262	66/M	Moderate dementia
AG06869	60/F	Moderate dementia
AG05810	79/F	Severe dementia

Coriell Institute for Medical Research.
