# Corrigendum to “Slower Dynamics and Aged Mitochondria in Sporadic Alzheimer's Disease”

**DOI:** 10.1155/2021/1643631

**Published:** 2021-01-14

**Authors:** Patricia Martín-Maestro, Ricardo Gargini, Esther García, George Perry, Jesús Avila, Vega García-Escudero

**Affiliations:** ^1^Centro de Biología Molecular “Severo Ochoa” (UAM-CSIC), Nicolás Cabrera, 1. Cantoblanco 28049 Madrid, Spain; ^2^Centro de Investigación Biomédica en Red de Enfermedades Neurodegenerativas (CIBERNED), Valderrebollo, 5, 28031 Madrid, Spain; ^3^Brain and Mind Research Institute, Weill Cornell Medical College, Cornell University, 407 E 61st St. 1300 York Avenue, New York, NY 10065, USA; ^4^University of Texas at San Antonio, One UTSA Circle, San Antonio, TX 78249-0667, USA; ^5^Departamento de Anatomía, Histología y Neurociencia, Facultad de Medicina, UAM, Arzobispo Morcillo, 4, 28029 Madrid, Spain

In the article titled “Slower Dynamics and Aged Mitochondria in Sporadic Alzheimer's Disease” [1], the following acknowledgement was omitted in error, “pTRE-Tight-MitoTimer was purchased from Addgene where it was originally deposited by Dr. Roberta Gottlieb (Addgene plasmid # 50547; http://n2t.net/addgene:50547; RRID: Addgene_50547.”
